# Supplementary material for: PI3Kγ stimulates a high molecular weight form of myosin light chain kinase to promote myeloid cell adhesion and tumor inflammation
Source: Nat Commun. 2022 Apr 1;13:1768. doi: 10.1038/s41467-022-29471-6 (PMC8975949; doi:10.1038/s41467-022-29471-6)
Supplement: Supplementary file 1 — Supplementary Information [file 41467_2022_29471_MOESM1_ESM.pdf]

Supplementary Information

PI3Kγ stimulates a high molecular weight form of myosin light chain kinase to promote myeloid cell adhesion and tumor inflammation

Michael C. Schmid, Sang Won Kang, Hui Chen, Marc Paradise, Anghesom Ghebremedhin, Megan M. Kaneda, Shao-ming Chin, Anh Do, D. Martin Watterson, and Judith A. Varner

Supplementary Figure 1

Supplementary Figure 2

Supplementary Figure 3

Supplementary Figure 4

Supplementary Figure 5

Supplementary Figure 6

Supplementary Figure 7

Supplementary Figure 8

Supplementary Figure 9

Supplementary Figure 1

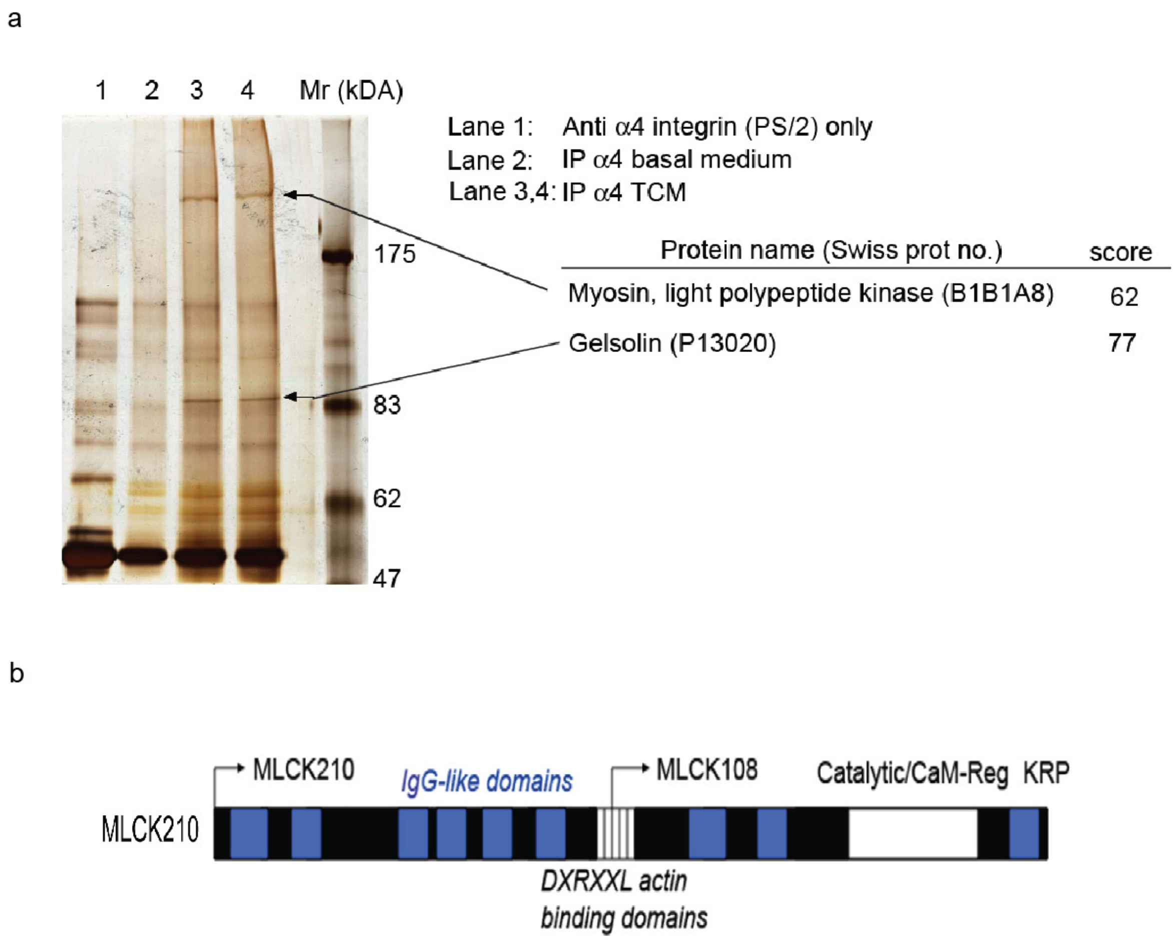

Supplementary Figure 1: *Mlck210* is an integrin α4 associated protein.
(a) Silver stained gel of integrin α4 immunoprecipitates of basal and TCM stimulated myeloid cells. from which 80 and 210 kDa proteins were excised for tandem mass spectrometry peptide sequencing. (b) Schematic depicting the structure of MLCK210.

Supplementary Figure 2

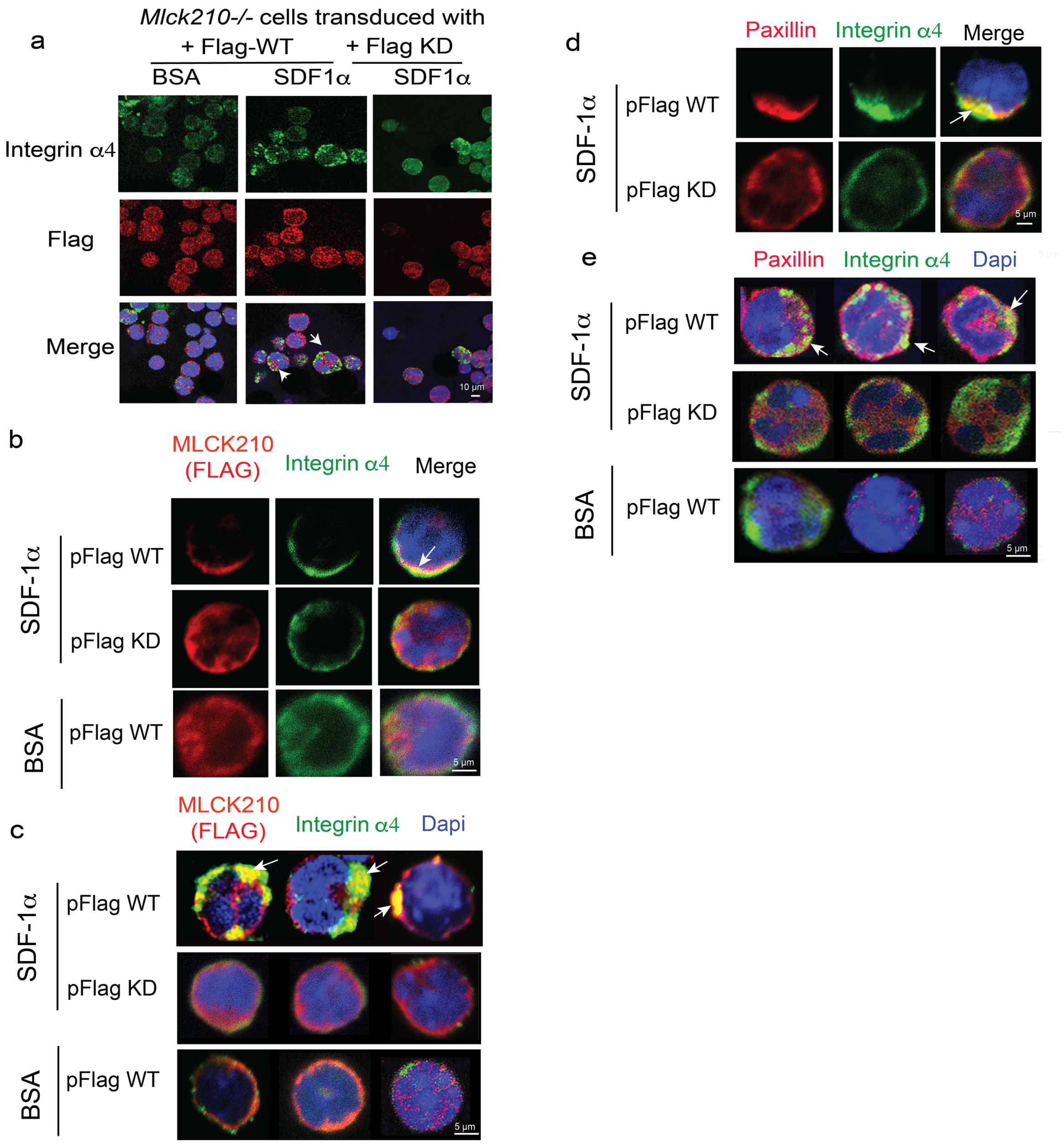

Supplementary Figure 2: *Mlck210* regulates integrin α4 clustering.

(a-c) Immunofluorescence detection of integrin α4 (green) and Flag-tagged MLCK210 (red) and integrin α4 (green) and Rap1 (red) in *Mlck210-/-* myeloid cells that were incubated with SDF-1α and BSA coated beads. (d-e) Immunofluorescence detection of integrin α4 (green) and Rap1 (red) in *Mlck210-/-* myeloid cells that were incubated with SDF-1α and BSA coated beads. Arrows indicate notable areas of overlap of MLCK210 or Rap and integrin α4. Nuclei were detected with Dapi (blue).

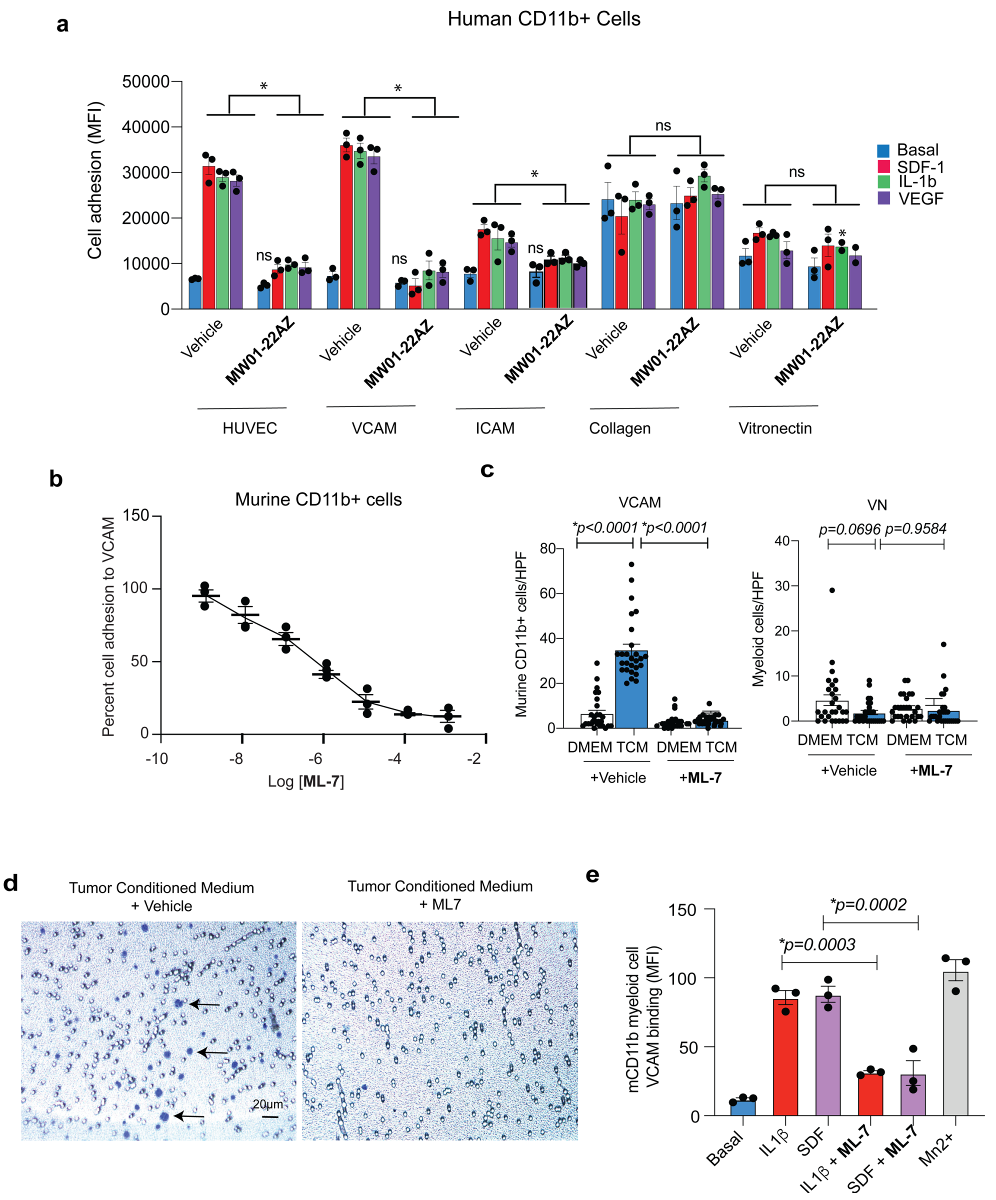

Supplementary Figure 3: MLCK210 is required for myeloid cell integrin α4 activation.
(a) Adhesion of **MW01-022AZ** or vehicle treated cytokine-stimulated myeloid cells to HUVEC (n=3 Vehicle vs **MW01-022AZ**); (**p=0.0132* basal; **p=0.0005* SDF-1α, **p<0.0001* IL-1β, **p=0.0003* VEGF-A); VCAM-1(*p=0.1335* basal; **p=0.0001* SDF-1α, **p=0.0006* IL-1β, **p=0.0003* VEGF-A); ICAM-1 (*p=0.2489* basal, **p=0.003* SDF-1α, **p=0.0088* IL-1β, **p=0.0014* VEGF-A); collagen (*p=0.8763* basal, *p=0.3579* SDF-1α, *p=0.0763* IL-1β, *p=0.2369* VEGF-A); or vitronectin (*p=0.3866* basal, *p=0.3545* SDF-1α, *p=0.2* IL-1β, *p=0.7157* VEGF-A). (b) Titration of the MLCK inhibitor **ML-7** on myeloid cell adhesion to VCAM-1 (n=3). (c) Effect of **ML-7** on myeloid cell migration toward basal medium or LLC tumor conditioned medium on VCAM or Vitronectin (VN)-coated substrates (n=27). (d) Images of migrated cells from c. (e) Effect of **ML-7** or vehicle on VCAM-Fc binding (MFI) to basal, IL-1β, SDF-1α and Mn^{2+} stimulated murine myeloid cells (n=3). Data are presented as mean values +/- SEM. Significance determined by one-way Anova with Tukey's multiple comparisons.

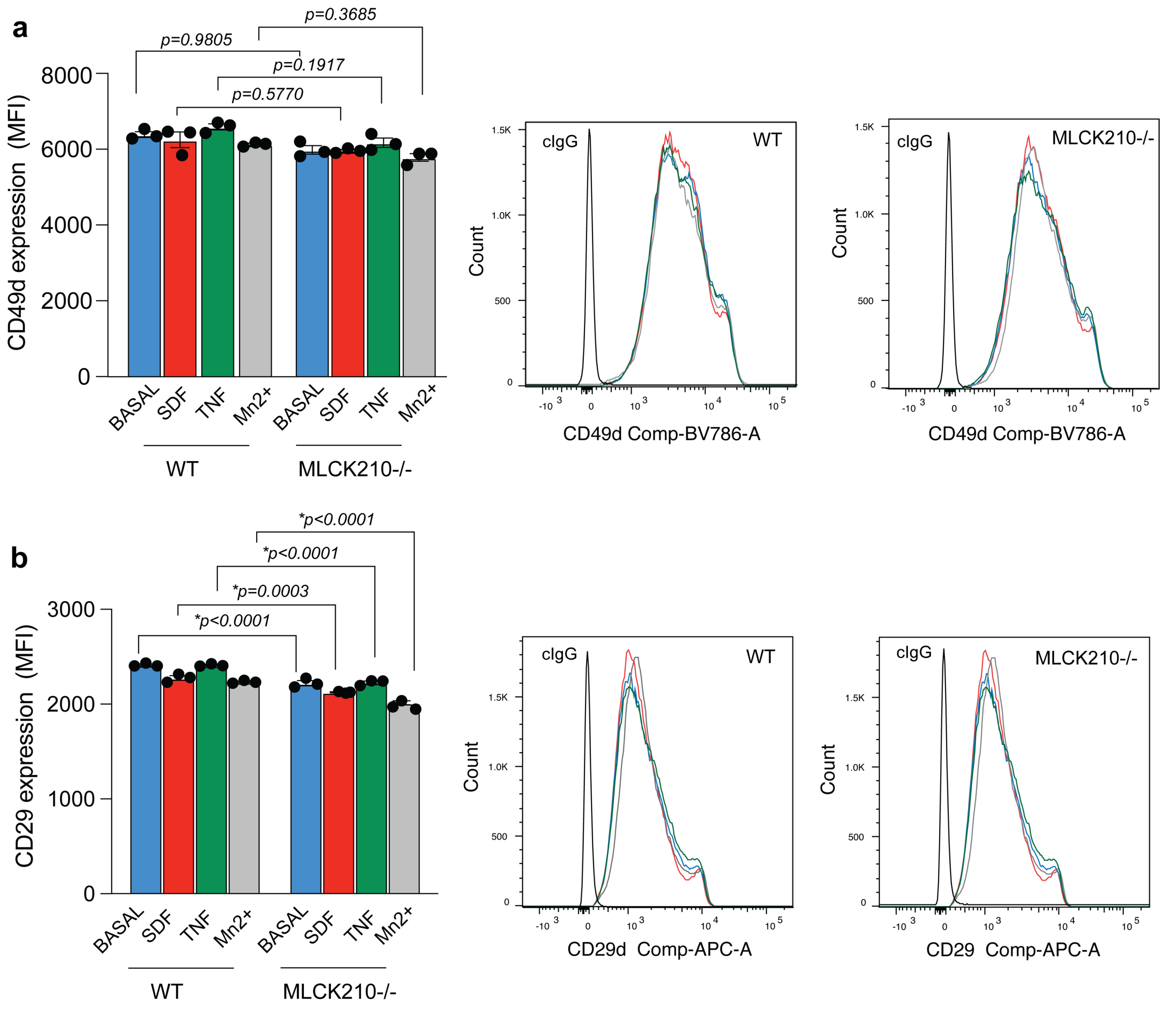

Supplementary Figure 4: Integrin α4β1 expression levels in cytokine-stimulated cells.
(a-b) Flow cytometry profiles and mean fluorescence intensity of (a) integrin α4 (n=3) and (b) integrin β1 subunit (n=3) cell surface expression levels in basal, SDF-1, TNF and Mn2+ stimulated WT and *Mlck210-/-* myeloid cells. Data are presented as mean values +/- SEM. Significance determined by one-way Anova with Tukey's multiple comparisons.

Supplementary Figure 5

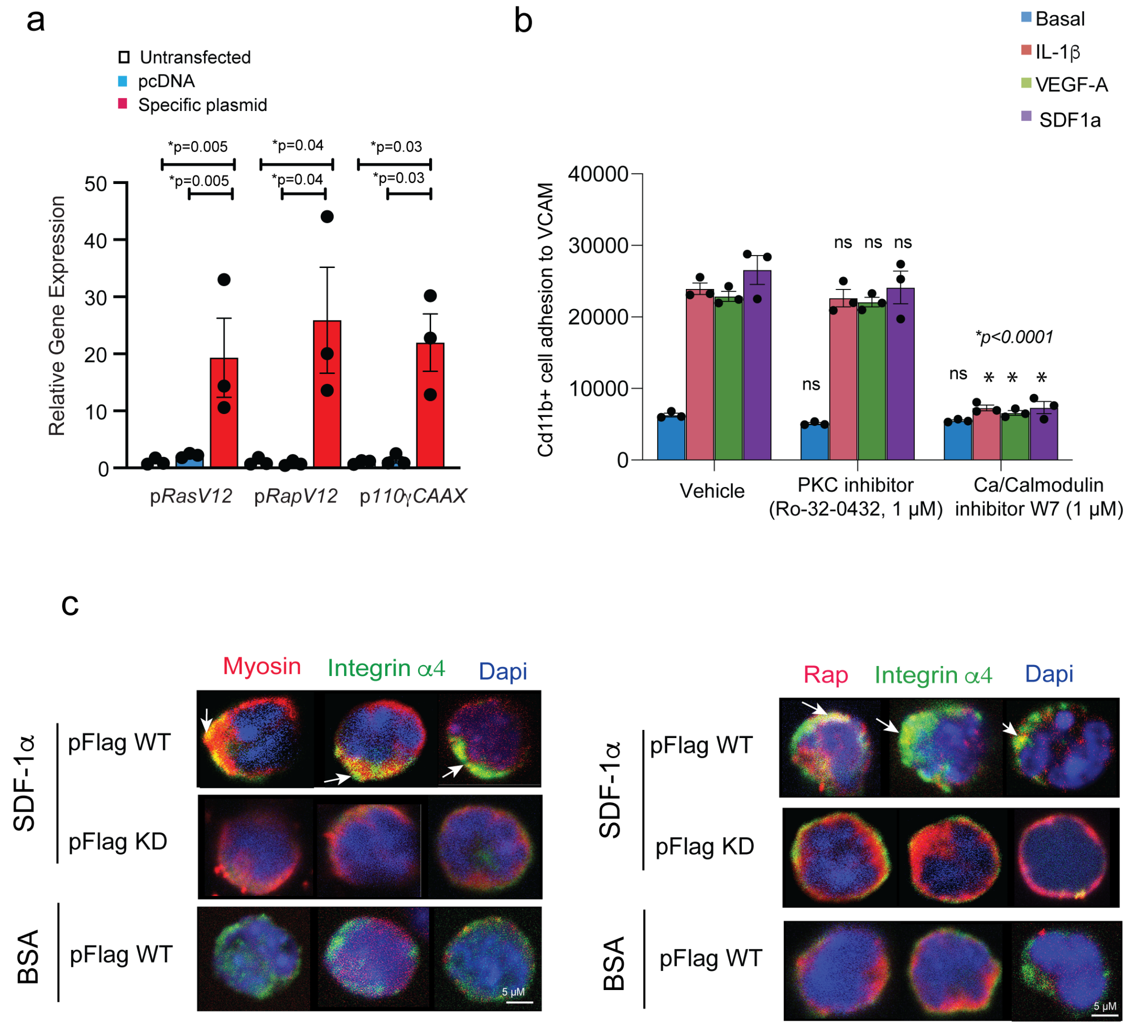

Supplementary Figure 5: Catalytically activity of MLCK210 is required for integrin α4 association with the cytoskeleton.

(a) RT-PCR detection of RasV12, RapV12 and p110γCAAX expression in myeloid cells that were transiently transfected with pRasG12V, pRapG12V or p110γCAAX (n=3). (b) Effect of the PKC inhibitor R0-32-0432, the calcium/calmodulin inhibitor W7 and vehicle on cytokine stimulated myeloid cell adhesion to VCAM-1 (n=3; ns indicates not significant; **p<0.0001*). *p=0.7138* basal, vehicle vs Ro-32-0432; *p=0.6621* IL-1β, vehicle vs Ro-32-0432; *p=0.852* VEGF-A, vehicle vs Ro-32-0432, *p=0.2529* SDF-1α, vehicle vs Ro-32-0432; *p=0.9626* basal, vehicle vs W7; **p<0.0001* IL-1β, vehicle vs W7; **p<0.0001* VEGF-A, vehicle vs W7, **p<0.0001* SDF-1α, vehicle vs W7. (c) Immunofluorescence analysis of co-localization of integrin α4 and MLCK210, myosin, Rap1, and Paxillin in WT but not catalytically inactive (kinase dead, KD) transfected *Mlck210-/-* myeloid cells. Scale bar = 5 µm. Data are presented as mean values +/- SEM. Significance determined by one-way Anova with Tukey's multiple comparisons.

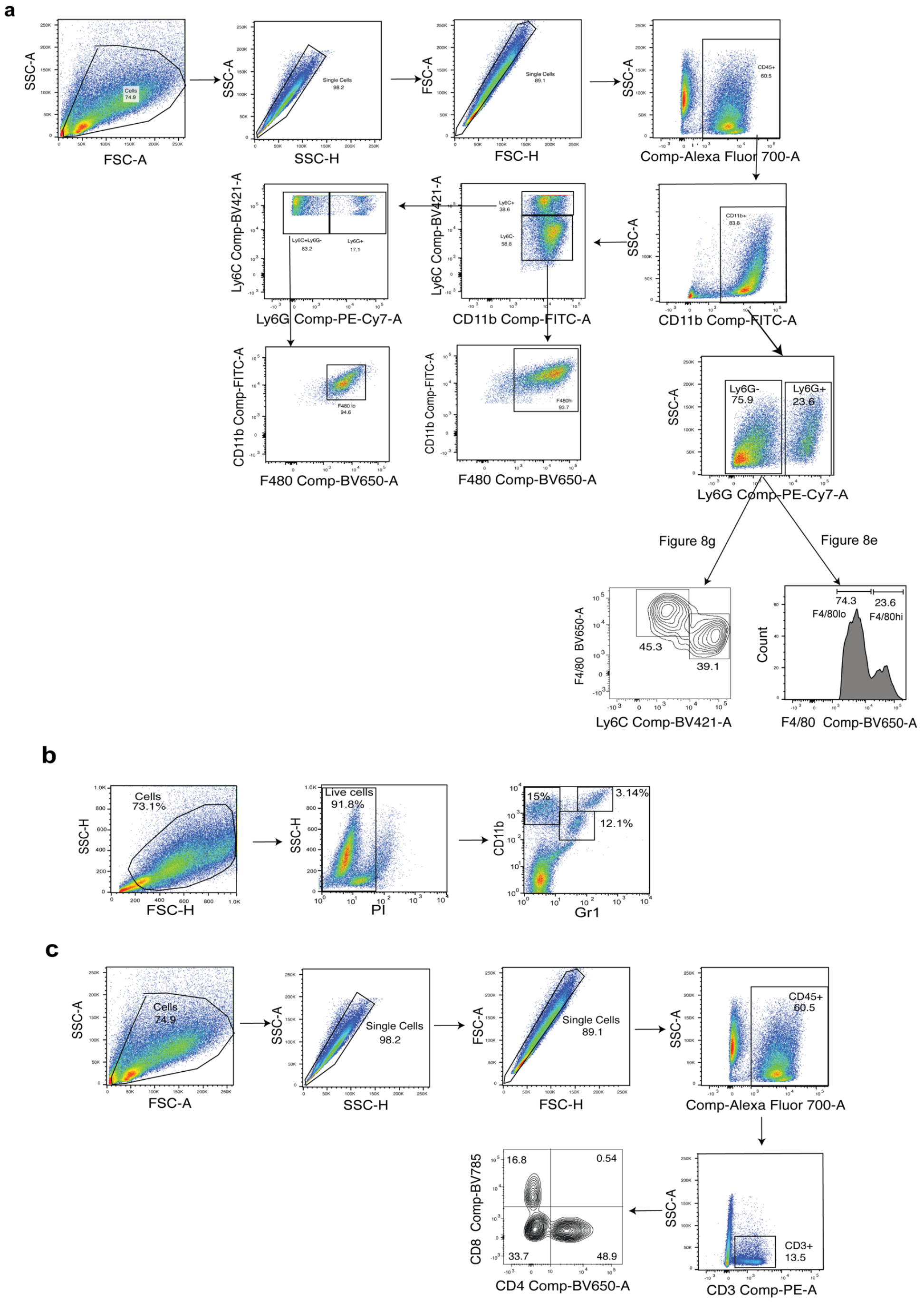

Supplementary Figure 6: Facs gating strategies.

(a) Flow cytometric gating scheme for the characterization of myeloid cells in tumors gated for BUV395, anti-CD45 AF-700, anti-FITC-CD11b, PE-Cy7 Anti-Ly6G, BV421 Anti-Ly6C, SBV670 F4/80. (b) Flow cytometric gating scheme for the characterization of myeloid cells in tumors gated for PI, CD11b and Gr1. (c) Flow cytometric gating scheme for the characterization of T cells gated for BUV395, anti-CD45 AF-700, anti-CD3 PE, Anti-CD4 BV650 and anti-CD8 BV785.

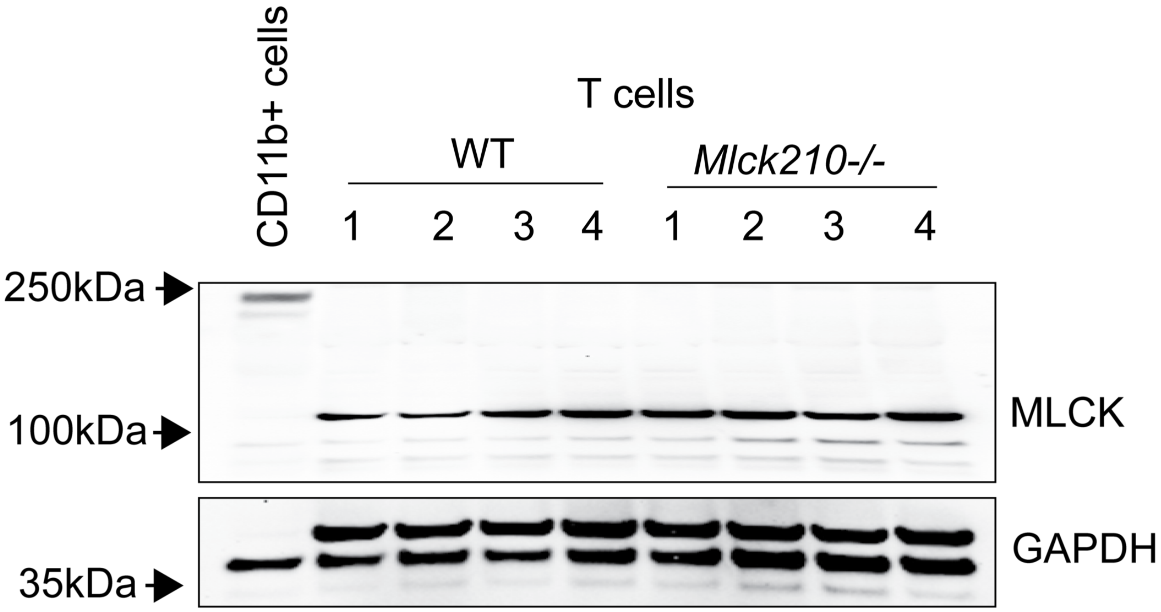

Supplementary Figure 7: MLCK210 expression in myeloid cells and T cells.
Immunoblot of MLCK210 and loading control GAPDH in purified bone marrow derived CD11b+ cells from WT mice and purified splenic T cells from WT and *Mlck210-/-* mice (n=4).

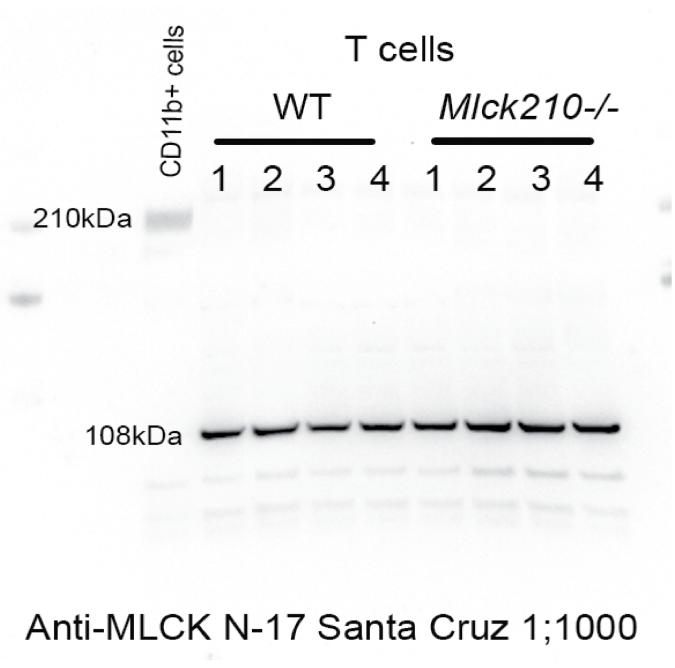

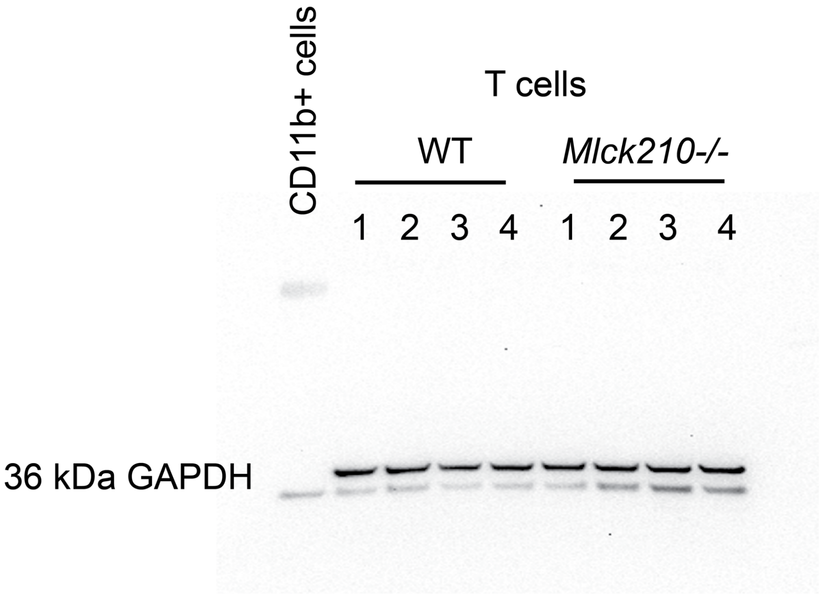

Supplementary Figure 8: Original gels, related to Supplementary Figure 7
Images of original gels of CD11b+ myeloid cells and T cells from WT and *Mlck210-/-* mice immunoblotted for MLCK (upper gel) and for GAPDH (lower gel).

a

Tandem mass spectrometry

Protein name (swiss prot no. or TrEMBL no)	Precursor ion (expt)	Precursor ion (calc)	Score	Residue number	Peptide sequence
Myosin, light polypeptide kinase (B1B1A8)	1824.32	1824.92	62	33-49	VSSMPLTEAPAFILPPR
	1600.41	1600.34	40	1239-1554	SSLPPVLGTESDATVK

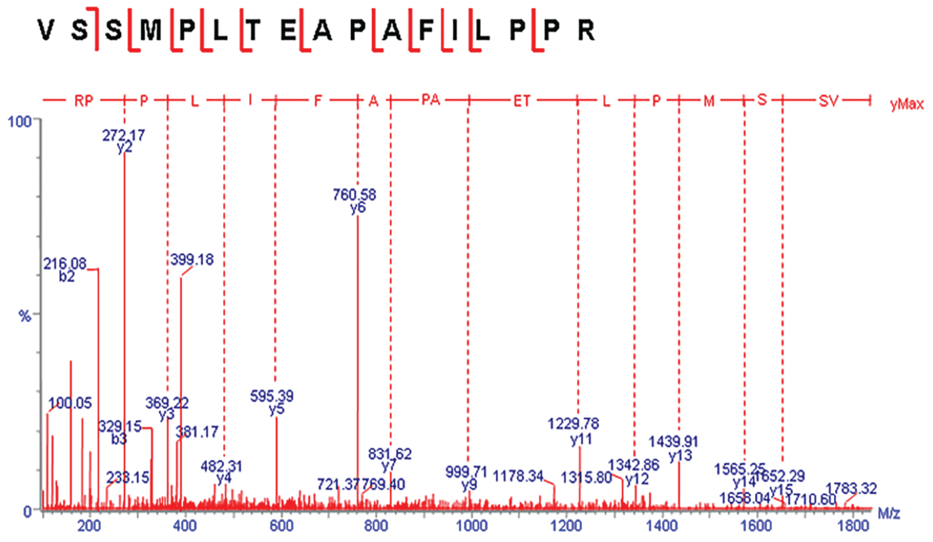

b

Protein name (swiss prot no. or TrEMBL no)	Precursor ion (expt)	Precursor ion (calc)	Score	Residue number	Peptide sequence
Gelsolin (P13020)	1334.69	1334.68	43	583-595	SGALNSNDAFVLK
	1554.76	1664.77	77	712-726	DSQEEEKTEALTSAK

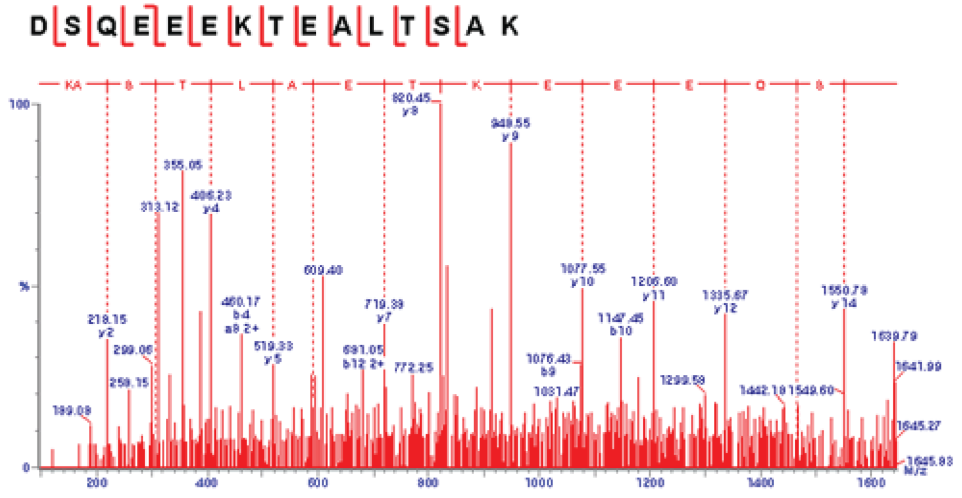

Supplementary Figure 9: Proteomics data

(a) 210 kDa silver stained gel band: Table of peptide sequences and spectra indicate protein is a high molecular weight isoform of myosin light chain kinase MYLK (MLCK210)

(**b**) 85 kDa silver stained gel band: Table of peptide sequences and spectra indicate protein is Gelsolin
